# Decreasing the indications for radical nephrectomy: a study of multifocal renal cell carcinoma

**DOI:** 10.3389/fonc.2012.00084

**Published:** 2012-08-06

**Authors:** Maximiliano Sorbellini, Gennady Bratslavsky

**Affiliations:** ^1^Urology Research and Consultancy,Miami Beach, FL, USA; ^2^Department of Urology, State University of New York Upstate Medical University,Syracuse, NY, USA

**Keywords:** multifocality, multifocal RCC, bilateral RCC, nephron-sparing surgery, partial nephrectomy, outcomes

## Abstract

Multifocal renal cell carcinoma (RCC) has been reported in 5–25% of cases worldwide. Although management of patients with multifocal RCC has not been clearly defined, presence of multifocal renal masses in one kidney and a normal contralateral kidney has often been considered a reason for performing radical nephrectomy. This study reviews the world literature to provide an accurate estimate of the prevalence of multifocal RCC and evaluates the oncologic outcomes of multifocal RCC after exclusion of patients with known hereditary and familial renal syndromes. A PubMed search of the literature was performed for articles in the English language using the following terms for the query: “multifocal RCC,” “multifocality and RCC,” “multicentric RCC,” or “bilateral RCC.” The references of the published articles were also reviewed for additional publications. Articles that did not specifically exclude patients with familial RCC or known hereditary RCC syndromes were excluded for estimation of multifocality prevalence and oncologic outcomes. After applying our exclusion criteria, nine articles were selected and form the basis of the current analysis. Weighted averages were used to calculate the prevalence of multifocality. Multifocal RCC was found in 6.8% of cases (373 of 5433 patients). Ipsilateral multifocality was found in 6.8% of cases. Bilateral multifocality was found in 11.7% of cases. Of all cases reported in this study, only 10% underwent partial nephrectomy. The rest of the study cohort underwent radical nephrectomy. The review of the literature showed that the use of nephron-sparing techniques in patients with multifocal disease did not compromise oncologic outcomes, despite the need for reoperation in certain cases. In conclusion, multifocal RCC remains a prevalent entity. Most clinicians still prefer to perform radical nephrectomies in these patients despite proven equivalent oncologic outcomes compared to nephron-sparing techniques. Urologists should be aware of these data when proposing treatment options to patients with multifocal RCC.

## INTRODUCTION

Identifying patients with multifocal renal cell carcinoma (RCC) is of major importance due to its influence on the treatment strategy, timing, and treatment modality used in such patients. Management of multifocal RCC has been a source of multiple debates for several reasons. First of all, it remains one of the relative contraindications to partial nephrectomy (Campbell et al., 2007) as it causes many urologists to think twice before embarking on a nephron-sparing approach, either due to technical considerations or oncologic concerns (Vogelzang, 2006). Second, radical nephrectomy would prevent the patient from potentially requiring a future repeat renal surgery for new or recurrent lesions in the same renal unit; a procedure known to be technically challenging. Third, in the absence of specific genetic studies to identify the clonal origin of every lesion, some may feel uncomfortable assigning ipsilateral or contralateral recurrences as *de novo* satellite tumors rather than intra-renal metastases. Finally, the relative paucity of reported outcomes for patients treated for multifocal RCC has not allowed for a wide acceptance of the nephron-sparing approaches as a preferred treatment modality.

Multifocal RCC has been reported in 5–25% of cases in the world literature (Kinouchi et al., 1999; Wunderlich et al., 1999). This incongruent variability in values is likely due to a variety of reasons. First, some of those studies have not excluded all patients with hereditary or familial RCC, known to be multifocal in nature (Delakas et al., 2002; Klatte et al., 2007; Linehan et al., 2009; Sargin et al., 2009). Second the quality of imaging modalities as well as radiologic evaluation used in different eras or locations may have contributed to the variability in detection of multifocal RCC found by different groups (Schlichter et al., 2000). Third, the pathological assessment of specimens has also varied in the different reports. Some investigators examined the renal surface only, others examined the kidney after stripping its capsule, while others examined the kidneys after they had been serially sectioned. Finally, the absence of accepted criteria used to define multifocality may have also resulted in variations of the estimated prevalence of multifocal RCC in the literature.

In the present report, we aim to examine differences in multifocality definitions and detection strategies used in published studies to identify the prevalence of “sporadic” multifocal RCC. We also examine oncologic outcomes of surgeries for sporadic multifocal RCC.

## METHODS

### SYSTEMATIC REVIEW OF THE LITERATURE

A PubMed search of the literature was performed independently by each author for articles in the English language using the following terms for the query: “multifocal RCC,” “multifocality and RCC,” “multicentric RCC,” or “bilateral RCC.” The references of the published articles were also reviewed for additional publications. We excluded articles that did not specifically exclude patients with familial RCC or known hereditary renal cancer syndromes, and those articles in which the prevalence for multifocal RCC could not be determined. After applying our exclusion criteria, nine articles were selected and these form the basis for the current analysis. The oncologic outcomes were then tabulated from the articles that met our inclusion criteria. Inclusion and exclusion criteria were decided before the literature search was initiated.

For the purpose of this study, multifocality was defined as the presence of two or more tumors present in the same renal unit, regardless of histology or timing of detection in the absence of extra-renal metastatic disease. The prevalence of multifocal RCC was calculated using weighted averages after dividing the number of cases with multifocality by the number of total cases with RCC (solitary and multifocal RCC).

## RESULTS

The prevalence of non-hereditary sporadic multifocal RCC was calculated to be 6.8% (**Table 1**). The occurrence of bilateral multifocal tumors was found to be 11.7%. As shown in **Table 2**, a subset analysis of our cohort shows that metastasis-free survival and overall-survival are similar to those reported by other series (**Table 3**). Furthermore, oncologic outcomes are similar for patients treated for solitary as well as for multifocal tumors (in sporadic population). Finally, **Table 3** shows excellent renal functional preservation in the setting of multiple surgeries on the same renal unit (in hereditary population).

**Table 1:** List of peer reviewed articles included for analysis.

Reference	Serial section?	Total cases	Total multifocal	RN (%)	PN (%)	Ipsilateral multifocal (%)	Bilateral multifocal (%)
Tsivian et al. (2010)	Yes	560	81 (14.4%)	560	0	81 (14.4%)	n/a
Crispen et al. (2008b)	n/a	1113	60 (5.4%)	599 (53.8%)	514 (46.2%)	60 (5.4%)	8/37 (21%)
Krambeck et al. (2008)	n/a	140	n/a	114 (81.4%)	26 (19.6%)	n/a	11 (7.85%)
Richstone et al. (2004)	Yes	1071	57 (5.3%)	1071	0	57 (5.3%)	28 (2.6%)
Dimarco et al. (2004)	n/a	2373	130 (5.4%)	2373	0	130 (5.4%)	n/a
Bilen et al. (1999)	Yes	40	4 (10%)	40	0	4 (10%)	n/a
Kinouchi et al. (1999)	Yes	124	23 (18.5%)	124	0	23 (18.5%)	n/a
Oya et al. (1995)	Yes	108	7 (6.5%)	108	0	7 (6.5%)	n/a
Whang et al. (1995)	Yes	44	11 (25%)	44	0	11 (6.5%)	n/a
			Weighted average:			Weighted average:	Weighted average:
			6.82%			6.82%	11.72%

**Table 2:** Oncologic outcomes for the study cohort.

Reference	Number of cases	Number of multifocal kidneys	Median follow-up (year)	Metastasis-free survival at 5 years (%)		Cancer-specific survival at 5 years (%)		Overall-survival at 5 years (%)		Contralateral tumor-free survival at 5 years (%)	
				Solitary tumors	Multifocal tumors	Solitary tumors	Multifocal tumors	Solitary tumors	Multifocal tumors	Solitary tumors	Multifocal tumors
Crispen et al. (2008b)	1113	60	6.1	97.6	96.5	98.7	96.2	84.4	84	99.1	94.4
Krambeck et al. (2008)	140	140	6.1	n/a	n/a	n/a	90.5 (95.8 if NSS)*	n/a	n/a	n/a	n/a
Richstone et al. (2004)	1071	57	3.4	73.2	71.5	n/a	n/a	79.3	75.2	84.9	82.5

*n/a, not available.*

* CCS at 5 years for cases treated with radical nephrectomy or nephron-sparing surgery are 90.5 and 95.8%, respectively.

**Table 3:** Functional outcomes post-nephron-sparing techniques in hereditary multifocal RCC.

Reference	Number	Median follow-	Change in serum
	of cases	up (month	creatinine (mg/dl)
Bratslavsky et al. (2008)	13	25	+0.2
Johnson et al. (2008)	51	56	+0.19
Herring et al. (2001)	62	30	+0.01
Liu et al. (2010)	25	60	+0.2

## DISCUSSION

We found that the prevalence of sporadic multifocal RCC was 6.8% (**Table 1**). This number is intriguing as the 6.8% rate of multifocality found in this study correlates well with the historic incidence of ipsilateral recurrence after partial nephrectomy of 4–9% (Kinouchi et al., 1999; Crispen et al., 2008a; Peycelon et al., 2009). This appears to suggest that many of the lesions that have been considered recurrences or intra-renal metastasis in the past might have just been incipient multifocal lesions not identified pre-operatively or during the actual surgical procedure. Notably, many studies have shown that most satellite lesions, in excess of 60%, are not detected by modern pre-operative imaging studies (Baltaci et al., 2000; Crispen et al., 2008b; Krambeck et al., 2008).

### DEFINING MULTIFOCAL RCC

One of the major problems in the study of multifocal RCC is precisely how to define multifocality itself. What defines a multifocal tumor? Is it simply the presence of another lesion within the same renal unit (Miyake et al., 1998; Junker et al., 2002)? Must the second lesion by necessity be malignant? Must there be a minimum amount of parenchyma in between primary and satellite lesions in order for multifocality to exist (Sargin et al., 2009)? Must all lesions share the same histology? Does the presence of a contralateral tumor also define multifocal disease (Bani-Hani et al., 2005)? Does the contralateral kidney need to contain multiple tumors for it to be multifocal? And is the multifocality on one kidney related to the multifocality on the opposite kidney? Furthermore, how can we even know if a second lesion within the same renal unit, or on the contralateral kidney for that matter, is a new *de novo* lesion unrelated to the primary tumor, or a local recurrence or metastasis from the primary lesion?

Unfortunately, without performing a specific genetic study designed to evaluate the clonal origin of each mass found it is often difficult to determine whether a second renal lesion implies that patients have multifocal, recurrent, or metastatic disease (Miyake et al., 1998; Junker et al., 2002; Blute et al., 2003). Because of our inability to answer these questions, in the present study, we defined multifocality in a broad manner in order to incorporate all the data available to us. Thus, we defined multifocality as the presence of two or more tumors present in the same renal, regardless of histology or timing of detection, in the absence of extra-renal metastatic disease in patients not known to have familial disease or hereditary syndromes. We acknowledge that some patients in our study may nevertheless be part of obscure or yet-to-be-discovered RCC syndromes. This is because the diagnosis of hereditary or familial RCC is not always straight forward and while certain syndromes are well know to all urologists, others, such as familial renal syndromes seen in patients with tuberous sclerosis or succinate dehydrogenase deficiency, for example, are not. In terms of histology, from our earlier studies of hereditary renal tumors we know that multiple tumors of various histologies can coexist within the same renal unit. We also know that the same genetic abnormality can lead to both benign and malignant renal lesions within the same kidney and that these lesions can also be found bilaterally and metachronously. For example, patients with von Hippel–Lindau (VHL) syndrome are prone to develop bilateral multifocal recurrent clear cell RCC; patients with hereditary papillary RCC (HPRCC) develop bilateral papillary type 1 RCC; hereditary leiomyomatosis RCC (HLRCC) patients tend to form aggressive papillary type 2 RCC and Birt–Hogg–Dubé (BHD) patients may develop a whole spectrum of tumors from benign oncocytomas to chromophobe RCC to aggressive clear cell RCC (Toro et al., 2008). Furthermore, as our knowledge of the genetic basis of renal neoplasias grows we keep finding new types of hereditary syndromes, such as the less well understood syndromes of tuberous sclerosis and succinate dehydrogenase B deficiency (Sudarshan and Linehan, 2006).

### HISTORICAL PERSPECTIVE AND CURRENT APPROACH TO MULTIFOCAL RCC

Ever since Robson’s 1969 article (Robson et al., 1969) suggested that radical nephrectomy should be the treatment of choice for patients with renal tumors, that practice has become standard of care, in particular for kidneys harboring multifocal RCC. Of note, Robson et al.’s (1969) conclusion was based on a report of outcomes of a few selected patients. During the next 30–40 years, in large part due to the knowledge gained through patients who underwent partial nephrectomies for imperative indications, Robson’s model has come under question. Initial reports about the technical feasibility of nephron-sparing procedures performed for small renal masses and subsequent reports of its oncologic efficacy, similar to that of radical nephrectomy, have slowly built a foundation for a wider utilization of partial nephrectomy (Russo et al., 2002). Additionally, with the understanding that RCC was not a single entity but a conglomeration of neoplastic processes, each with its own biological identity and aggressiveness (Kovacs et al., 1997), the field of urology has slowly realized that perhaps a one-operation-fits-all model was not the best way to approach every type of renal tumor. Thus, the indications for elective partial nephrectomy began to expand (Russo et al., 2002), resulting in a much larger cohort of cases to be analyzed and compared to outcomes of radical nephrectomy.

We now know that, despite previous teachings, not only tumors smaller than 4 cm but also larger than 4 cm are amenable to nephron-sparing techniques (Russo, 2007). We also know that stage-by-stage the oncologic outcomes seem to be similar between partial nephrectomy and radical nephrectomy cohorts (Bratslavsky et al., 2008; Johnson et al., 2008; Breau et al., 2010; Liu et al., 2010). Furthermore, recent data have confirmed that patients undergoing radical nephrectomies for RCC are at higher risk not only of developing renal insufficiency (Huang et al., 2006; Sorbellini et al., 2006) but more ominously, to die of non-cancer-related causes, such as cardiovascular events, compared to patients undergoing nephron-sparing techniques (Huang et al., 2009). Additionally, data from patients treated with multiple partial nephrectomies for hereditary and familial RCC syndromes indicate that the oncologic outcome is not altered in those patients when they undergo multiple repeated partial nephrectomies (Gupta et al., 2010). Finally, National Cancer Institute data on repeat surgical interventions on a same kidney indicate that kidneys can withstand multiple injuries due to surgical procedures and renal function is usually well-preserved (**Table 3**), preventing patients from having to undergo dialysis or renal transplantation (Bratslavsky et al., 2008; Gupta et al., 2010).

Despite all this information, indications for partial nephrectomy still need to be enumerated and radical nephrectomies are still favored by a large number of urologists even for small solitary renal tumors of less than 4 cm in maximum diameter (Russo, 2006). In fact, at the present time there are no NCCN Guidelines regarding multifocal RCC tumors. Perhaps a better treatment approach for sporadic multifocal RCC would be performing the same type of surgery as for patients with familial or hereditary RCC syndromes, i.e., resection of all detectable renal lesions via nephron-sparing techniques as they become apparent and reach a certain size (Walther et al., 1999). The latter is supported by the fact that: (a) there is no oncologic benefit to performing radical nephrectomy versus partial nephrectomy, (b) there are no data proving that newly discovered post-surgical lesions are indeed recurrences or metastases after partial nephrectomy, (c) at least 20% of RCC lesions are either benign or indolent in nature, and (d) the data showing that losing a renal unit puts patients at higher risk not only of developing renal insufficiency but also of death from non-cancer-related causes (Crispen et al., 2008a). Finally, as the data presented in this study show, a significant number of patients with multifocal RCC will have renal lesions in the contralateral kidney, possibly necessitating surgery at a later date, increasing their chances of becoming anephric, had radical nephrectomy previously performed.

### CLINICAL IMPLICATIONS

Identifying patients at higher risk for metastatic disease and those who are likely to fail local therapy is indeed of paramount importance. Nevertheless, treating patients with overkill surgeries under the presumption that more is better puts patients at risk not only for renal insufficiency but also for cardiovascular disease and death from non-cancer-related causes (Sorbellini et al., 2006; Huang et al., 2009). Radical nephrectomy may not only be unnecessary in these patients but it may also prevent those patients from future treatments (e.g., targeted therapies) that require a normal renal function to qualify for them. Although we strongly advocate appropriate surgery for local control of renal lesions, with negative surgical margins, the reader should also be aware of recent data showing that positive surgical margins do not jeopardize survival outcomes in patients undergoing nephron-sparing techniques (Yossepowitch et al., 2008). Therefore, we encourage surgeons to attempt nephron-sparing surgery whenever technically feasible and, in cases of multifocal disease.

### LIMITATIONS OF THE STUDY

The present study has several limitations. It is possible that some of the patients included in our analysis were undiagnosed hereditary RCC cases. We only reported results on patients treated surgically and may have potentially missed those with metastatic disease that were not included in the original publications.

## CONCLUSION

Multifocal RCC remains an underappreciated and misunderstood entity with a prevalence of at least 6%. Bilateral multifocal disease is found in 11.7% of cases. The presence of multiple renal tumors in one kidney appears to increase the risk of having renal lesions in the contralateral kidney. Twenty percent of multifocal RCC are benign or indolent in nature. Stage-by-stage, oncologic outcome appears similar for patients treated with radical or partial nephrectomy. Resection of multiple renal lesions from the same renal unit in a single setting is feasible and may allow for excellent preservation renal function. Radical nephrectomy, in the context of RCC, appears to increase the risk of renal insufficiency and death from unrelated causes. Nephron-sparing techniques should thus be considered whenever technically feasible, in particular in cases of multifocal RCC.

## Conflict of Interest Statement

The authors declare that the research was conducted in the absence of any commercial or financial relationships that could be construed as a potential conflict of interest.
